# Bidirectional long short-term memory with CRF for detecting biomedical event trigger in FastText semantic space

**DOI:** 10.1186/s12859-018-2543-1

**Published:** 2018-12-21

**Authors:** Yan Wang, Jian Wang, Hongfei Lin, Xiwei Tang, Shaowu Zhang, Lishuang Li

**Affiliations:** 1 School of Computer Science and Technology, Dalian University of Technology, Dalian, China; 2 School of Information Science and Engineering, Hunan First Normal University, Changsha, China

**Keywords:** Biomedical events, Trigger detection, Bidirectional LSTM, CRF, Semantic space, FastText

## Abstract

**Background:**

In biomedical information extraction, event extraction plays a crucial role. Biological events are used to describe the dynamic effects or relationships between biological entities such as proteins and genes. Event extraction is generally divided into trigger detection and argument recognition. The performance of trigger detection directly affects the results of the event extraction. In general, the traditional method is used to address the trigger detection as a classification task, as well as the use of machine learning or rules method, which construct many features to improve the classification results. Moreover, the classification model only recognizes triggers composed of single words, whereas for multiple words, the result is unsatisfactory.

**Results:**

The corpus of our model is MLEE. If we were to only use the biomedical LSTM and CRF model without other features, the F-score would reach about 78.08%. Comparing entity to part of speech (POS), we find the entity features more conducive to the improvement of performance of detection, with the F-score potentially reaching about 80%. Furthermore, we also experiment on the other three corpora (BioNLP 2009, BioNLP 2011, and BioNLP 2013) to verify the generalization of our model. Hence, F-scores can reach more than 60%, which are better than the comparative experiments.

**Conclusions:**

The trigger recognition method based on the sequence annotation model does not require initial complex feature engineering, and only requires a simple labeling mechanism to complete the training. Therefore, generalization of our model is better compared to other traditional models. Secondly, this method can identify multi-word triggers, thereby improving the F-scores of trigger recognition. Thirdly, details on the entity have a crucial impact on trigger detection. Finally, the combination of character-level word embedding and word-level word embedding provides increasingly effective information for the model; therefore, it is a key to the success of the experiment.

## Background

In biomedicine, understanding the context of a biomedical event is significant in information extraction for existing biomedical literatures. In general, biological events are used to describe the dynamic interaction between biological entities, such as proteins and genes. Using NLP techniques, an event extraction system predicts relations between proteins/genes and the processes in which they take part.

The biomedical event extraction task has been successfully applied four times so far: BioNLP 2009 [1], BioNLP 2011 [2], BioNLP 2013 [3] and BioNLP-ST 2016 [4]. Whether in a phased system (e.g., UTurku system [5], EVEX system [6], EventMine system [7], et al) or a joint system (e.g., UMass system [8], FAUST system [9], et al.), the method put forward in these four share-tasks should contain two steps: the first step is trigger detection, and the second is argument detection. Obviously, trigger detection is an essential and crucial step in event extraction. The trigger detection’s performance will ultimately affect the result regarding event extraction.

In general, we would initially confirm if a word is a trigger, otherwise known as a binary classification task. Then, we would perform a multiple classification task by identifying the type of triggers. There are three traditional bases for methods of trigger detection: they can be based on statistic or dictionary [10, 11], on rule [12, 13], or on machine learning approaches [14–16]. In their work, Buyko et al. made use of a dictionary-based approach to detect triggers [10]. They first researched all triggers in the original GENIA corpus, and then had biology students sort them by category of events. Finally, the experts divided triggers and built parts of triggers into a dictionary. Because there are some limitations to this method, we found its precision was about 47%, and the F-scores were 46.66%. Cohen et al. used a rule-based approach to detect triggers [12]. First, they researched all triggers that have been annotated. Second, they made a statistical analysis of the frequency of all triggers, and found a higher frequency of words. Finally, they built a language model around the concept of “category” with these words. Despite the possibility of finding additional triggers for a rule-based approach, its rules are rather complex; tremendous amounts of time is required to make rules, and detection may be severely affected by imperfect or inappropriate rules. As previously stated, the accuracy of this method could reach approximately 71% in accuracy; yet, its recall rate was disappointing, with F-scores of only 22.7%. The approach for biomedical events extraction is based on machine learning in share-tasks. In general, trigger detection of the machine learning-based approach includes automatically learning features and training classifiers of triggers. Therefore, it is regarded as a task of classification. The advantage of the machine learning-based method is to save manpower, resources and time, and its recognition effect is better compared to other methods. The machine learning-based approach generally uses SVM, CRF, or maximum entropy models, all of which require more complex feature engineering. With the extensive application of machine learning, a deep learning network in natural language also revealed better results. Li et al. used a neural network model to train word embedding as a basic feature [17]. This was used for biomedical event extraction, which provided a new direction for simplifying the feature design.

It is relatively rare for researchers to study trigger detection as an independent project. In the traditional method, trigger detection is normally seen as a multi-classification problem; therefore, to promote the recognition performance of triggers, external tools are often used to analyze the original corpus and help obtain more valuable features. Because the task of classification relies on more complicated feature engineering, it will lead to high development costs in new languages and a new corpus. Consequently, in 2011 [18], Collobert et al. proposed a neural network framework which uses word embedding and convolution operations, and obtained favorable outcomes on four tasks of NLP sequence annotation. These outcomes indicated that the framework has a good generalization capability and learning ability, and does not depend on cumbersome features of the project. Based on analysis, this paper will address trigger detection as a sequence annotation, and will result in a method of trigger detection based on bidirectional long short-term memory (LSTM) and conditions random field (CRF) in FastText semantic space applied on a multi-level event extraction corpus (MLEE) [19]. The results of this paper will be compared to other models.

## Methods

### Basic progress

**Fig. 1:**
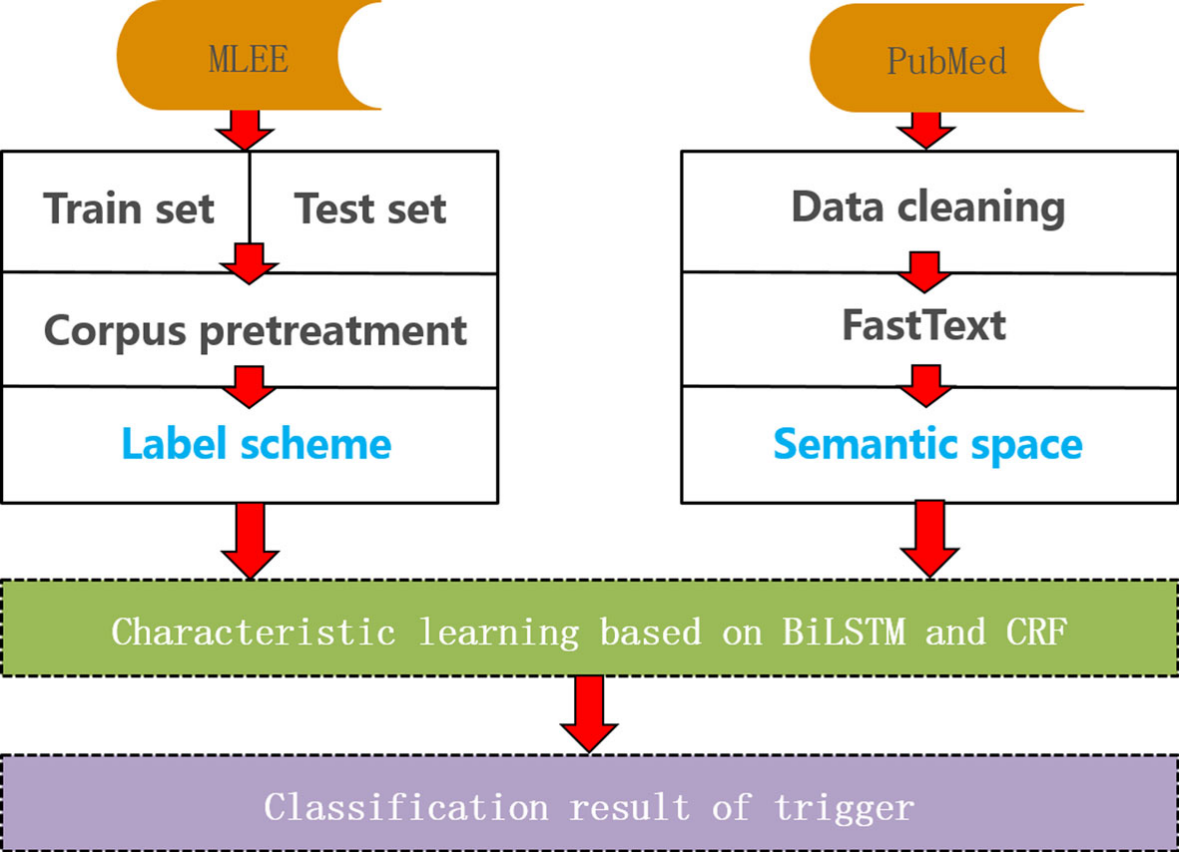
The basic process of trigger detection

Figure 1 shows that the basic process of trigger detection includes three parts.

First, the MLEE corpus is preprocessed by providing each word with an IBO label, called the label scheme. Then, a word-embedding training tool (FastText) is used to build semantic space for different words from the PubMed corpus. Finally, the bidirectional LSTM model is established to help in understanding the historical features of each word. Concurrently, the CRF model is used to obtain sentence-level label information and ensure the relevance and accuracy of the label sequence.

### Label scheme

In biomedicine, detecting triggers is a challenging problem. The words or phrases of the same form can be used as triggers for different event types. Therefore, it is difficult to obtain the type of triggers and the range of triggers (how many words constitute a trigger).

**Fig. 2:**
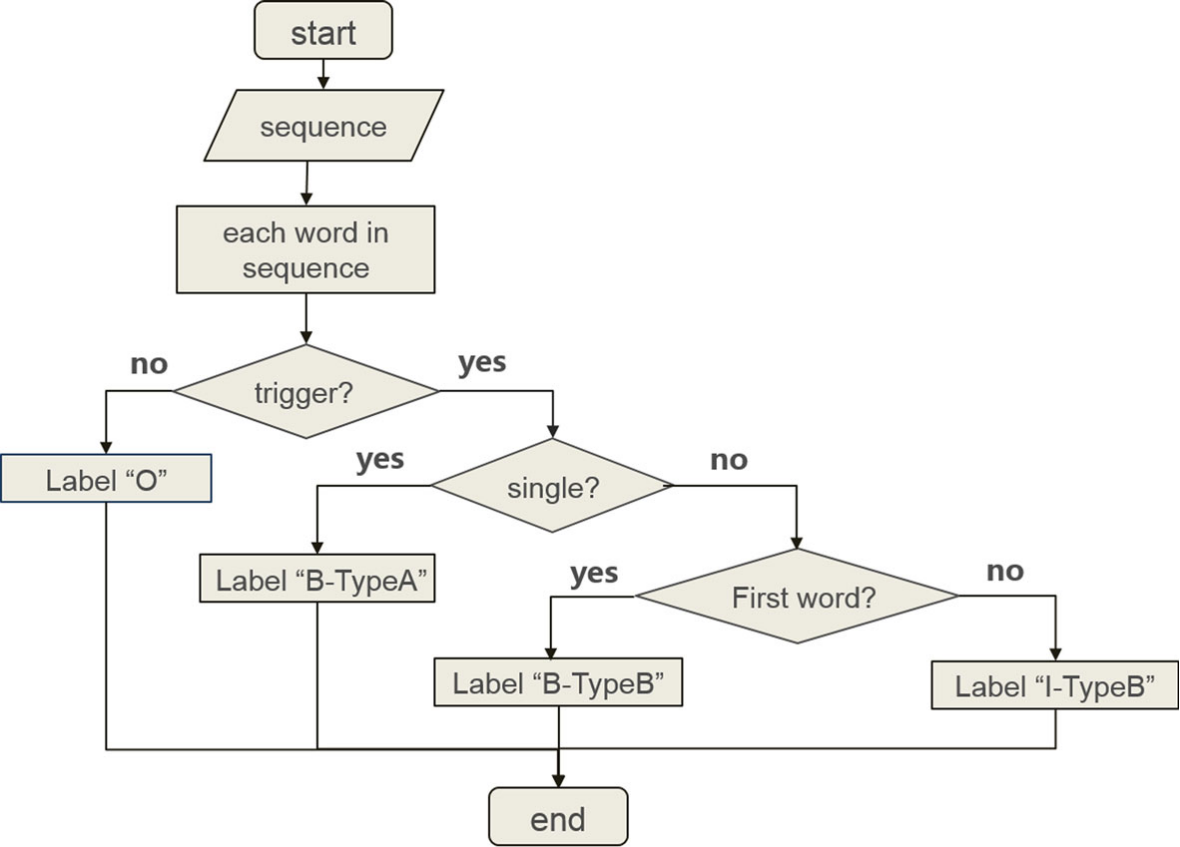
The processing flow of “BIO” label mechanism

In trigger detection, approximately 10% of triggers are composed of multiple words in the MLEE corpus. If the task is classified, only the single word can be recognized. Therefore, how are multi-word triggers detected? This paper proposes a “BIO” label mechanism with annotated triggers which consist of a word or a phrase to help solve the issue of trigger detection. As shown in Fig. 2, the letter “I” represents “inside”, the letter “B” represents “begin”, and the letter “O” represents “out”. Thus, the flowing rules were created: if a word is a trigger, it will be marked as “B-type”; if a phrase is a trigger, the first token within the trigger will be marked as “B-type” and the other tokens within the trigger will be marked as “I-type”. Other words that are not triggers will be marked as “O”.

For example, a flowing sequence from the corpus is given as follows: “VEGF plays a key role in the angiogenic response that occurs with chronic bradycardia”. There are two triggers: one is “plays a key role”, whose event type is “Regulation”; and the other is “angiogenic”, whose event type is “Blood_vessel_development”. Table 1 shows the annotation information.

**Table 1:** The example of annotation information

Word	Label
VEGF	O
Plays	B-Regulation
A	I-Regulation
Key	I-Regulation
Role	I-Regulation
In	O
The	O
Angiogenic	B-Blood_vessel_development
Response	O
That	O
Occurs	O
With	O
Chronic	O
Bradycardia	O
.	O

### Build semantic space

Text language is different from picture or audio information, because a semantic relationship exists between the words. However, traditional one-hot coding is random and sparse, and does not have any correlation information. For example, one-hot encoding was used for “China” and “Beijing”, with results of 5178 and 3987, respectively. These results indicated that there is no correlation between two values. Nevertheless, it is common knowledge that Beijing is the capital of China. Therefore, vector expressions were used to solve this problem.

The vector space model can convert words into vector expressions of consecutive values, and map similar words into similar vector spaces. This is called semantic space.To improve the performance of trigger detection, we use FastText tools [20, 21] to build semantic space.

**Fig. 3:**
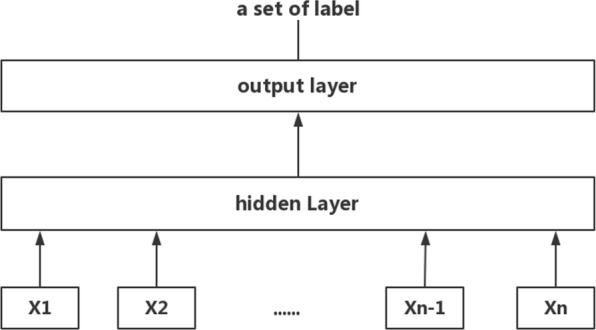
The structure of FastText model

FastText, as the name suggests, is a simple and efficient text classification tool for a standard multicore CPU trained on more than one billion words in less than 10 min. Figure 3 shows a linear model with rank constraint. This model is like the CBOW model of word2vec. The only difference between FastText and CBOW is that the middle word is replaced by a label. The traditional linear classifier will spend expensive to compute complexity on a large number of classes, and the computational complexity is *O(kh)*, where *k* is the number of classes and *h* is the dimension of the text representation. However, FastText uses a hierarchical softmax based on the Huffman coding tree, wherein computational complexity drops to *O*(*hlog*_2_(*k*)).

To enrich the word vector, the FastText model considers the morphology of the word and proposes a “sub-word” model. This model assesses each word as a bag of character n-grams (in practice, n greater or equal to 3 and smaller or equal to 6). Adding special boundary symbols 〈 *and* 〉 at the beginning and at the end of words, allow for the ability to distinguish between prefixes and suffixes from other character sequences. Similarly, the word itself will be included in the set of its n-gram. For example, if we want to represent the word “regulation” as 3-gram, it will be shown as: 〈*re, reg, egu, gul, ula, lat, ati, tio, ion, on*〉.

And the special sequence is 〈*regulation*〉. the word “regulation” will thus be represented as the sum of the 3-gram vectors.

FastText has several advantages: high training speed, applicability to large-scale corpus, and the efficiency for low-frequency words. Therefore, its performance is better than Word2Vec.

### Feature learning

**Fig. 4:**
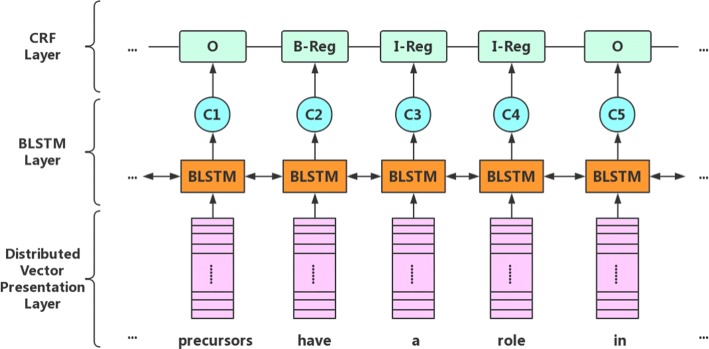
The bidirectional LSTM and CRF mode

**Fig. 5:**
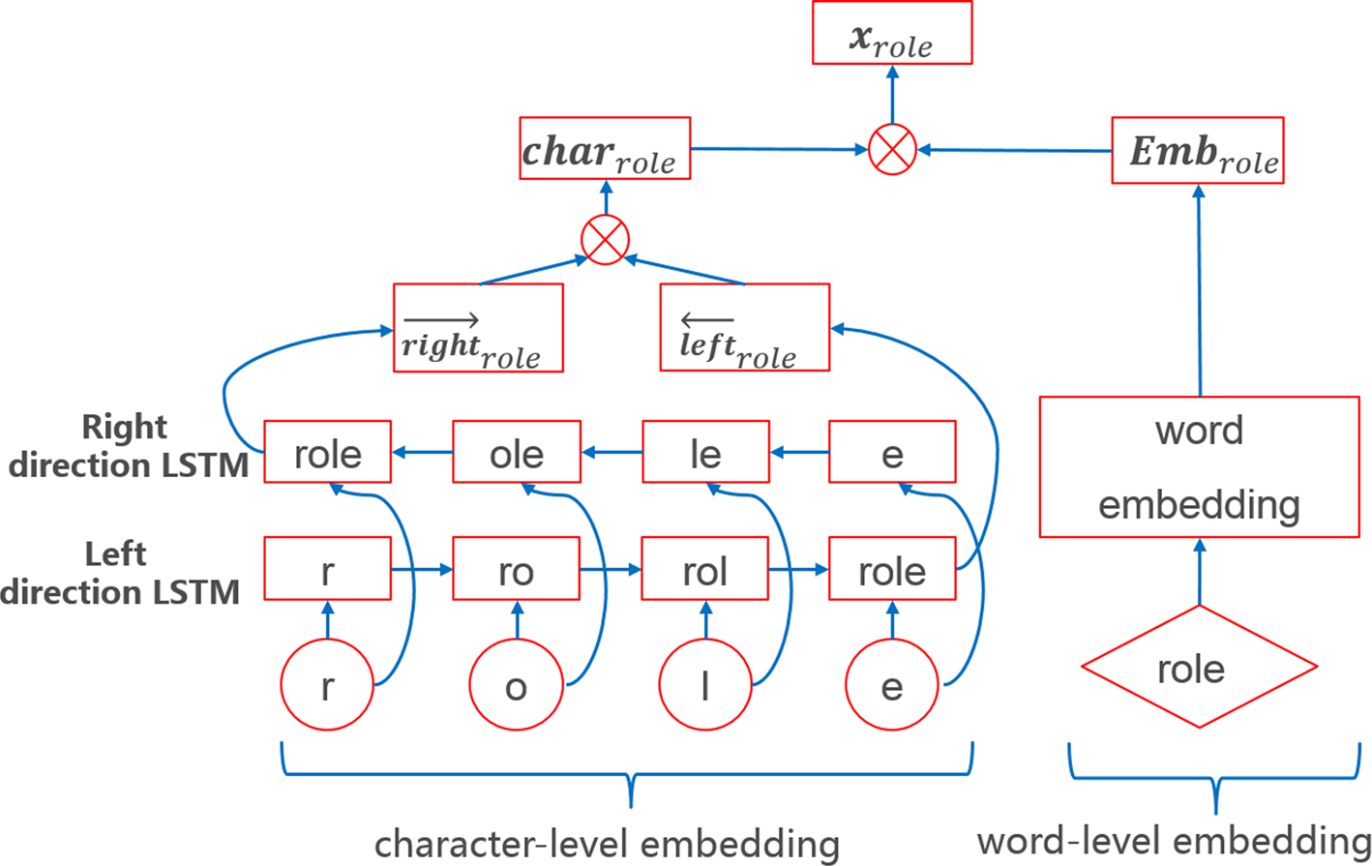
The distributed vector presentation layer

This paper used the bidirectional LSTM and CRF model to detect triggers in corpus, which has three layers: distributed vector presentation layer, bidirectional LSTM layer, and CRF layer – all of which are shown in Fig. 4. For the distributed vector presentation layer, two different kinds of word embedding presentations were used to identify triggers. The first is based on word-level representation from a semantic space. If the word does not appear in the semantic space, we will randomly initialize the word vector. The second is based on character-level representation used in supervised learning to obtain results from the MLEE corpus. The greatest advantage of character-level representation is its ability to express the word prefix and suffix information in considering the information of word shape. As shown in Fig. 5, bidirectional LSTM was used to train character-level embedding and was combined with word-level embedding, thus resulting in more valid word vector as input for the next layer. For example, if we want to train a word, we will input this word into two distinct directions of LSTM. In left-direction LSTM, we can obtain the forward information of this word, whereas in right-direction LSTM, the backward information of this word can be utilized. We use Eq. (1) to concatenate it to different direction information; therefore, we can get $\phantom {\dot {i}\!}{char}_{{word}_{i}}$, otherwise known as character-level embedding. Simultaneously, we refer to the table on semantic space generated by word-embedding tools, which could result in the word-level embedding of this word. In the end, concatenating the character-level embedding and word-level embedding information with Eq. (2) will yield a final word vector.

1 $$\begin{array}{@{}rcl@{}} \mathbf{char}_{{word}_{i}} = [ \vec{right}_{{word}_{i}} ; \vec{left}_{{word}_{i}}]. \end{array} $$

2 $$\begin{array}{@{}rcl@{}} \mathbf{x}_{{word}_{i}} = [ \mathbf{char}_{{word}_{i}} ; \mathbf{Emb}_{{word}_{i}}]. \end{array} $$

Where $\vec {left}_{{word}_{i}}$ represents the character-level word vector through the left direction LSTM and $\vec {right}_{{word}_{i}}$ represents another character-level word vector through the right direction LSTM. $\phantom {\dot {i}\!}{\boldsymbol {Emb}}_{{word}_{i}}$ represents the word-level embedding.

**Fig. 6:**
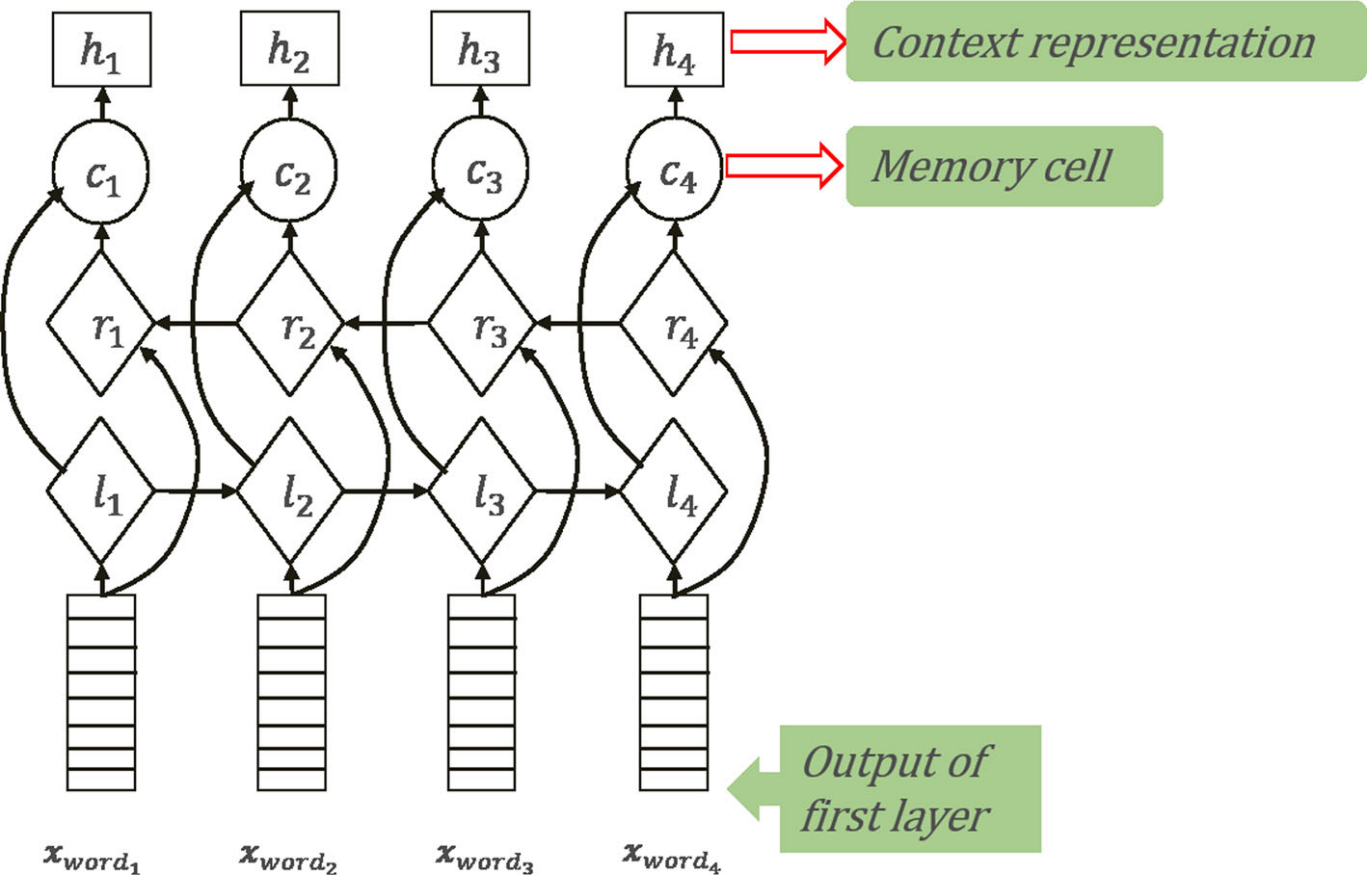
The bidirectional LSTM layer

The second layer is the bidirectional LSTM layer. As demonstrated by Fig. 6, $\phantom {\dot {i}\!}x=(x_{{word}_{1}},x_{{word}_{2}},...,x_{{word}_{n}})$ is the result of the first layer. We use it as the input of the second layer, which is similar to the process of character-level representation. However, the only significant difference is the timing of the bidirectional LSTM regarding the recording of information. In this layer, it is the intact information – not the segmental character information of each word – that will be remembered. The memory cell could record the significant information of the specific direction of LSTM. In the end, this results in the context feature sequence ***h***=(***h***_***1***_,***h***_***2***_,...,***h***_***i***_,...,***h***_***n***_), where ***h***_***i***_ is the context representation of *word*_*i*_ that can be obtained in Eq. (3).

3 $$\begin{array}{@{}rcl@{}} \boldsymbol{h}_{\boldsymbol{i}} = [\boldsymbol{fordward}_{h_{i}} ; \boldsymbol{backward}_{h_{i}}] \end{array} $$

When there is a correlation between the labels, we can use the condition random field (CRF) to learn the global information effectively. Thus, the third layer, CRF layer, will help in improving the performance of trigger detection. We take advantage of the sequence result ***h*** to predict a sequence of labels *y*=(*y*_1_,*y*_2_,...*y*_*n*_). We define a score matrix P, which size is *n*×*k*, where k the number of is different labels, and *P*_*i*,*j*_ denotes the score of the *j*^*th*^ label of the *i*^*th*^ word. Therefore, we use Eq. (4) to define its score.

4 $$\begin{array}{@{}rcl@{}} {f}(\boldsymbol{h},\boldsymbol{y})=\sum\limits_{i=0}^{n}\boldsymbol{M}_{{y_{i}},{y_{i+1}}} + \sum\limits_{i=1}^{n}\boldsymbol{P}_{i,{y_{i}}} \end{array} $$

Where M a matrix of transition is in reference to scores, and *M*_*i*,*j*_ represents the score of a transition from the label *i* to label *j*. The first part of the equation represents the transfer feature, and the latter represents the state feature. When given a feature sequence h of the specific instance and a label sequence y, we can then use Eq. (5) to maximize the target function by the CRF label.

5 $$\begin{array}{@{}rcl@{}} {f}(\boldsymbol{h},\boldsymbol{y})-\log\sum_{\tilde{y}\in{\boldsymbol{Y}_{h}}}\exp{{f}(\boldsymbol{h},\tilde{\mathbf{y}})} \end{array} $$

Where *Y*_*h*_ represents a set of all probable label sequences. Therefore, $\tilde {\mathbf {y}}$ is the predicted value, and **y** is the actual value

## Results and discussion

From the experiment, we drew two conclusions: the effectiveness of our model, and the generalization of this model. The model in this paper is an end-to-end system, which is better for achieving a multi-word-driven trigger detection task.

### Effective result

In Table 2, we compare the results without syntax information with Zhou [22] and Pyysalo [19]. The experiments are implemented in the MLEE corpus. According to the results, we discover F-scores to be 78.08 [23], which are better than those of the contrast experiment. Hence, this indicates that our model for trigger detection is effective. Different semantic spaces have distinct performance of detection. Therefore, to compare with other word-vector tools, we used three additional tools (Word2Vec, Doc2Vec, and Global vector) to build semantic space. In Table 3, it is evident that the performance of FastText is better compared to others. We deem that the n-gram of FastText considers morphology of the word, which thus pays more attention to the similarities in word composition. Accordingly, it can provide more effective information for the training of word embedding. Regarding sequence annotation tasks, there are several methods with which to experiment, such as CRF, LSTM, LSTM-CRF and bidirectional LSTM. Thus, this paper will use these methods to detect triggers with the same input as our model, with the semantic space of FastText. Table 4 demonstrates that the CRF model has a better recall rate; however, its accuracy and F-scores are inadequate. This is most likely due to that specific CRF model’s reliance on feature engineering. Nevertheless, our input is of the original sentence without any other linguistic information. As for the LSTM model, its accuracy and F-scores are much higher, although the recall rate is lower than that of the CRF model. Hence, a LSTM-CRF model combines the advantages of both LSTM and CRF, which would balance and improve the overall performance. Compared to LSTM models, a bidirectional LSTM model has greater memory capacity because of its two distinguish-direction memory information, evidently resulting having the second highest F-scores. If we utilize extra linguistics knowledge, such as part of speech, we can enhance the input feature, and improve the trigger detection results. Therefore, we developed a simple part-of-speech feature to verify our speculation. In addition, there is an abundance of entity information in the original corpus. How does entity information affect recognition performance? The specific results are shown in Table 5. According to the experimental results, we observed little effect POS features had on trigger detection. Nevertheless, entity information plays a key role in the process of detection. It is our belief that the event extraction is crucial in the relationship between triggers and entities. Thus, entity information could enhance valuable trigger information to improve the performance of trigger detection.

**Table 2:** Comparison of trigger detection performance with existing methods

Method	F-score(%)	Precision(%)	Recall(%)
Pyysalo [19]	75.67	70.65	81.46
Zhou [22]	77.82	74.85	81.04
Ours [23]	78.08	77.89	78.28

**Table 3:** Comparison of trigger detection performance with different word embedding

Semantic Space	F-score(%)	Precision(%)	Recall(%)
Random Embedding	73.72	76.36	71.25
Glove	74.84	79.70	70.54
Doc2Vec	76.03	78.78	73.47
Word2Vec	76.71	79.61	74.02
Ours(FastText)	78.08	77.89	78.28

**Table 4:** Comparison of trigger detection performance on different model

Model	F-score(%)	Precision(%)	Recall(%)
CRF	65.45	57.44	76.06
LSTM	72.60	78.40	67.61
LSTM-CRF	75.22	76.12	74.35
BLSTM	76.39	80.02	73.08
Ours(BLSTM-CRF)	78.08	77.89	78.28

**Table 5:** The result with features of entity and POS

Features	F-score(%)	Precision(%)	Recall(%)
Ours	78.08	77.89	78.28
Ours+POS	78.12	77.99	72.22
Ours+entity	79.58	80.58	71.57
Ours+POS+entity	80.64	75.28	76.86

### Generalizable results

In general, sequence annotation model has generalization ability. Therefore, to demonstrate the detection performance of our model, we applied the model to the BioNLP 2009, BioNLP 2011 and BioNLP 2013 corpus. The results are shown in Table 6. Although the results of detection are unsatisfactory, the F-scores could reach more than 60%. Because of the integrality of event extraction, recent experiments on trigger recognition were not found using these three corpora. However, by comparing Martinez [16], Vlachos [14], and Liu [24], the generalization of our model was found to be satisfying. In other words, the annotation model does not require complex feature engineering, which can be easily migrated to other corpora or tasks. All the experimental results have a range of floating. Since the results were unstable, we took the average of 10 experimental results and 0.5 as the floating range.

**Table 6:** The result of different corpus

Corpus	systems	F-score(%)	Precision(%)	Recall(%)
BioNLP 2009	Ours	63.01	68.21	58.55
	Martinez [16]	60.10	70.20	52.60
BioNLP 2011	Ours	66.81	68.44	65.26
	Vlachos [14]	58.98	66.76	52.82
BioNLP 2013	Ours	64.66	63.08	66.33
	Liu [24]	50.95	54.22	48.06

## Conclusion and future work

In this paper, we developed a method of trigger detection based on bidirectional LSTM and CRF by impleneting only the simplest features such as POS and entity information. According the experimental results, we observed that the model without feature engineering can reach better results. Nevertheless, the simplest feature can help improve the performance of detection. In contrast to different corpora, our model has positive generalization ability, and the results of detection were all effective. Building semantic space is also crucial and important for success as is the full use of information of word shape to establish word-level embedding by N-gram of FastText., and its combination with character-level embedding to get more useful input in the distributed vector presentation layer. Compared to the classification task, trigger detection can be regarded as a sequence annotation task, to solve the issue of recognizing a multi-word trigger properly. In the future, we will try to use the attention mechanism to increase the weight of valid features, to improve the accuracy and F-scores of trigger detection.

## Abbreviations

CRF: Conditions random field
LSTM: Long short-term memory
MLEE: Multi-level event extraction
POS: Part of speech
